# Oxidative Stress-Related Parthanatos of Circulating Mononuclear Leukocytes in Heart Failure

**DOI:** 10.1155/2017/1249614

**Published:** 2017-11-09

**Authors:** Tamás Bárány, Andrea Simon, Gergő Szabó, Rita Benkő, Zsuzsanna Mezei, Levente Molnár, Dávid Becker, Béla Merkely, Endre Zima, Eszter M. Horváth

**Affiliations:** ^1^Department of Physiology, Semmelweis University, Budapest, Hungary; ^2^Heart and Vascular Center, Semmelweis University, Budapest, Hungary; ^3^Institute of Human Physiology and Clinical Experimental Research, Semmelweis University, Budapest, Hungary

## Abstract

**Background:**

The present study aims to examine the oxidative stress-related activation of poly(ADP-ribose) polymerase (PARP), a cause of parthanatos in circulating mononuclear leukocytes of patients with chronic heart failure (CHF), that was rarely investigated in the human setting yet.

**Methods:**

Patients with CHF (*n* = 20) and age- and body mass index-matched volunteers (*n* = 15) with a normal heart function were enrolled. C-reactive protein, N-terminal probrain-type natriuretic peptide (pro-BNP), plasma total peroxide level (PRX), plasma total antioxidant capacity (TAC), oxidative stress index (OSI), leukocyte lipid peroxidation (4-hydroxynonenal; HNE), protein tyrosine nitration (NT), poly(ADP-ribosyl)ation (PARylation), and apoptosis-inducing factor (AIF) translocation were measured in blood samples of fasting subjects.

**Results:**

Plasma PRX, leukocyte HNE, NT, PARylation, and AIF translocation were significantly higher in the heart failure group. Pro-BNP levels in all study subjects showed a significant positive correlation to PRX, OSI, leukocyte HNE, NT, PARylation, and AIF translocation. Ejection fraction negatively correlated with the same parameters. Among HF patients, a positive correlation of pro-BNP with PRX, OSI, and PARylation was still present.

**Conclusions:**

Markers of oxidative-nitrative stress, PARP activation, and AIF translocation in blood components showed correlation to reduced cardiac function and the clinical appearance of CHF. These results may reinforce the consideration of PARP inhibition as a potential therapeutic target in CHF.

## 1. Introduction

Heart failure as the outcome of a variety of cardiovascular diseases implies increasing burden for global healthcare systems [1]. Various pathophysiological conditions including ischemic heart disease, cardiomyopathies, myocarditis, long-standing arrhythmias, metabolic, hematologic, and genetic disorders may lead to impaired myocardial function [2, 3]. The progression of cardiovascular dysfunction to heart failure is a complex phenomenon and involves the activation of numerous secondary pathways.

Experimental and clinical studies provided a wide range of evidence of increased oxidative-nitrative stress in heart failure [2]. The integrity of cardiac myocytes is deteriorated by reactive species-damaging proteins, DNA, and membrane lipids. The increased oxidative-nitrative stress leads to the activation of various enzymes including matrix metalloproteinases and poly(ADP-ribose) polymerase-1 (PARP).

Poly(ADP-ribose) polymerase-1 (PARP) is the major isoform of an enzyme family, with multiple regulatory functions. Upon activation, mainly by DNA single-strand breaks, it cleaves NAD^+^ into nicotinamide and ADP-ribose, forms long branches of ADP-ribose polymers, and binds them to several nuclear target proteins (PARylation) [4]. PARP activation exerts its physiological and pathological effects via two principal mechanisms [5]. On one hand, (i) it PARylates acceptor proteins including histones, transcription factors, and the PARP itself. PARylation contributes to DNA repair and to the regulation of gene expression. The protein expression of various proinflammatory mediators (cytokines, chemokines, iNOS, and ICAM-1) is regulated by PARP at the transcriptional level. On the other hand, (ii) oxidative-nitrative stress-induced overactivation of PARP consumes NAD^+^ and consequently ATP, culminating in cell dysfunction and necrotic cell death [3].

Poly(ADP-ribosyl)ation was activated under critical situations with oxidative-nitrative stress-induced DNA damage and was shown to play a regulatory role in different cell death pathways, such as apoptosis, autophagy, necroptosis, and parthanatos [6].

During caspase-dependent apoptosis, PARP-1 is cleaved by caspases preventing further loss of NAD^+^ and providing energy for the apoptotic processes [7]. PAR accumulation can induce a form of caspase-independent death triggering the translocation of mitochondrial factors, including apoptosis-inducing factor (AIF). Pathogenic PAR polymer translocates to the mitochondria from the nucleus to mediate AIF release. AIF translocation to the nucleus promotes chromatin condensation and DNA degradation. This crosstalk between nucleus and mitochondria is the key point in special cell death pathway called parthanatos [8].

The present study aims (1) to examine parthanatos phenomenon (oxidative-nitrative stress, PARP activation, and AIF translocation) in blood components of patients with chronic heart failure, (2) to assess the possible correlation with the severity of the disease, and (3) to identify clinical parameters that may play a role in the development of parthanatos of circulating mononuclear cells in chronic heart failure.

## 2. Materials and Methods

### 2.1. Participants

20 patients between the age of 50 and 70 years with known chronic heart failure (CHF) having clinically stable cardiopulmonary state (NYHA II-III) were enrolled at the Heart and Vascular Center of Semmelweis University, Budapest, Hungary. Heart failure was diagnosed according to the actual guideline of the European Society of Cardiology (ESC) published in 2016. In accordance with the ESC guideline recommendations, transthoracic echocardiography was used to evaluate cardiac structure and function and to measure left ventricular ejection fraction (EF) according to the modified Simpson's rule (apical biplane method with tracing of endocardial borders). CHF diagnosis was verified by the reduced systolic function defined as ejection fraction (EF) below 40%. NYHA stages were also determined according to the ESC guideline. The etiology of the heart failure was divided to ischemic (having known ischemic heart disease (IHD), *N* = 9) or nonischemic (*N* = 11) origin, without further evaluation [9]. Transthoracic echocardiography performed within the last 3 months before enrollment was accepted if no significant change of patient condition was observed. In case of new symptoms, or lacking echocardiography in the previous 3 months, cardiac ultrasound was repeated. Volunteers matched by age, BMI, and cardiovascular risk factors (smoking, diabetes mellitus, hypertension, and IHD) with normal systolic function served as controls (*n* = 15). Exclusion criteria were insulin-treated diabetes mellitus, acute coronary syndrome, stroke or major surgical intervention in the past 3 months, acute or chronic inflammatory diseases, and neoplasm.

All procedures complied with the Helsinki Declaration of the World Medical Association and were approved by the institutional and regional ethics committee of Semmelweis University (7268-0/2011-EKU). Written informed consent was acquired from all participants.

### 2.2. Clinical Examinations

After enrollment, all participants completed an interviewer-assisted questionnaire that included demographic (age, gender, and lifestyle) and general medical (medication, known illnesses, and family history) inquiries. Current smoking was defined as one or more cigarette/day on a weekly average. Height and weight were measured on a calibrated stadiometer in light clothing. BMI was calculated as weight (kg)/height (m^2^). Transthoracic two-dimensional echocardiography was performed to detect left ventricular end diastolic diameter (LVEDD), left ventricular end systolic diameter (LVESD), and ejection fraction. Fasting blood samples were collected from all subjects for C-reactive protein (CRP), N-terminal probrain-type natriuretic peptide (pro-BNP), oxidative-nitrative stress, PARP activity, and AIF translocation measurements. CRP and pro-BNP levels were determined by immunochemical methods (Cobas Integra 400 Plus and Cobas E411 analyzers, resp.; Roche, Basel, Switzerland) at the Central Laboratory of the Heart and Vascular Center of Semmelweis University.

Ejection fraction, LVEDD, LVESD, and plasma pro-BNP levels of individuals were used to characterize cardiac function. Brain natriuretic peptide (BNP) is a member of a family of human natriuretic peptides. Before its activation, BNP is stored as the precursor, pro-BNP. After pro-BNP is secreted in response to volume overload resulting to myocardial stretch, it is cleaved to the biologically inactive N-terminal fragment and the biologically active hormone BNP. The 2 fragments are secreted into the plasma in equimolar mounts, and both have been clinically evaluated for use in the management of congestive heart failure. The secreted N-terminal fragment is usually referred to as simplified plasma pro-BNP. According to the newest guideline for the diagnosis and treatment of the heart failure of the European Society of Cardiology, elevated natriuretic peptides help to establish initial diagnosis, identifying those who require further cardiac investigation and patients with values below the threshold (125 pg/mL) for the exclusion of heart failure. Natriuretic peptides are strong predictors of mortality in chronic heart failure, and pro-BNP-guided therapies are shown to improve the outcome in heart failure [10–13].

### 2.3. Oxidative-Nitrative Stress and PARP Activation

Plasma total peroxide level (PRX) was measured by Oxystat kit (Biomedica, Vienna, Austria) according to the user's manual. Absorption was detected at 450 nm by BioTek Powerwave XS Spectrophotometer (BioTek, BioTek Instruments, Inc., Winooski, VT, USA). PRX measurements are available from 11 controls and 12 CHF patients. Total plasma antioxidant capacity (TAC) was measured by OxiSelect™ TAC Assay Kit (Cell Biolabs, San Diego) according to the user's manual. Absorption was detected at 490 nm by BioTek Powerwave XS Spectrophotometer. Results are given in CRE (*μ*mol copper reducing equivalent). TAC measurements are available from 15 controls and 19 CHF patients. Oxidative stress index (OSI) is given as the ratio of total peroxide and TAC. For the determination of cellular lipid peroxidation, protein tyrosine nitration, PARP activation, and AIF translocation, circulating mononuclear leukocytes, were isolated by gradient centrifugation (Histopaque-1077, Sigma-Aldrich, St. Louis, MO, USA). Cellular lipid peroxidation, protein tyrosine nitration, and PARP activation were estimated by immunohistochemical staining of methanol-fixed leukocyte smears with rabbit polyclonal anti-4-hydroxynonenal (HNE) (Abcam, Cambridge, UK; 1 : 200, overnight, 4°C), rabbit polyclonal antinitrotyrosine (NT) (Millipore, Temecula, CA, USA; 1 : 80, overnight, 4°C), mouse monoclonal anti-PAR (Tulip, West Point, PA, USA; 1 : 500, overnight, 4°C), and anti-AIF rabbit polyclonal (Millipore; 1 : 100, 4°C, overnight) antibodies. Measurements of AIF translocation are available in 11 patients with CHF and 13 control subjects.

Secondary labeling was achieved by biotinylated mouse and rabbit immunoglobulin-specific antibodies (Vector Laboratories, Burlingame, CA, USA). Avidin-horseradish peroxidase complex and brown-colored diaminobenzidine (DAB) (HNE) or black-colored nickel-enhanced DAB (NT, PAR, and AIF) (Vector) were used to visualize specific labeling. Blue-colored hematoxyilin (HNE) or red-colored Nuclear Fast Red (NFR) (NT, PAR, and AIF) (Sigma, MO, USA) was used as counterstain. Microscopic images of immunolabelled smears were acquired by Zeiss Imager A1 light microscope using 20x/0.45 and 40x/0.75 objectives, AxioCam MRc5 camera, and AxioVision Rel. 4.8 software (Carl Zeiss Microscopy GmbH, Jena, Germany). The ratio of the positive cellular area and total cellular area (HNE area (%) and NT area (%)) or the ratio of positive nuclear area and total nuclear area (PAR area (%) and AIF area (%)) analyzing at least 300 cells per smear was calculated by computer-based evaluation (MBF ImageJ, NIH, Bethesda, MA, USA).

### 2.4. Statistical Analysis

Sample size calculation was performed prior to the recruitment of study subjects based on two measured parameters: PRX and PAR areas. Standard deviation (SD) of these parameters were estimated using the data of our previous measurement among the healthy human subjects (*n* = 25) (unpublished data). The mean of PRX area was 313.4 *μ*mol/l with an SD of 118.5 *μ*mol/l, while the mean of PAR area was 12.45% with an SD of 8.72%. Calculating 80% power for *p* < 0.05, the sample size of 15 was found to be eligible to detect 125.94 *μ*mol/l and 9.3% change in PRX and PAR areas, respectively, which was decided to be sufficient to detect the expected change of 120 *μ*mol/l and 10% in CHF patients [14].

Results are expressed as mean ± SD for normally distributed variables and median (interquartile range) for nonnormally distributed variables. Variables that violated the normality assumption (CRP, pro-BNP, HNE area, NT area, PAR area, and AIF area) were log-transformed before using them in the statistical models. Participants' characteristics by study group were compared using two-tailed independent samples *t*-test. The distribution of categorical variables (gender, smoking, presence of diabetes mellitus, hypertension, or IHD) in the different groups was compared by chi-square test. The possible relationship between clinical parameters and oxidative-nitrative stress, PARP activation, AIF translocation in the study cohort, and among CHF patients was examined by Pearson's correlation test and linear regression models. In case of multiple significant correlations in the CHF group, multivariate linear regression models were implemented. Missing values were treated as missing. In all cases, *p* < 0.05 was considered significant (SPSS 22.0 and Graphpad Prism and StatMate softwares).

## 3. Results

### 3.1. Clinical Parameters and Anamnestic Data

In our study, 20 patients with chronic heart failure (CHF) and 15 controls were enrolled. The gender composition of the two groups was similar. Both age and body mass index of the two study groups were also comparable. There was no significant difference concerning the prevalence of hypertension, diabetes mellitus, ischemic heart disease, and smoking habit. The level of the inflammatory marker CRP was not significantly different in the study cohorts. Ejection fraction was significantly lower; LVEDD and LVESD were significantly higher in the diseased group. The level of pro-BNP was markedly increased in CHF patients. The median pro-BNP of control group is slightly elevated according to exclusionary cut-off point mentioned in the actual guideline of the European Society of Cardiology (125 pg/mL); however, these subjects showed no clinical signs of cardiac failure so the elevation of pro-BNP is most probably due to other conditions [9] (Table 1). In our CHF group, patients having IHD do not differ in any abovementioned parameters from their non-IHD mates except from significantly reduced LVEDD (CHF without IHD: 68.6 ± 10.6 mm versus CHF with IHD: 54.2 ± 3.5 mm, *p* < 0.05) and LVESD (CHF without IHD: 59.5 ± 9.9 mm versus CHF with IHD: 45.4 ± 3.8 mm, *p* < 0.05).

### 3.2. Oxidative, Nitrative Stress, PARP Activation, and AIF Translocation in Blood Components

**Figure 1:**
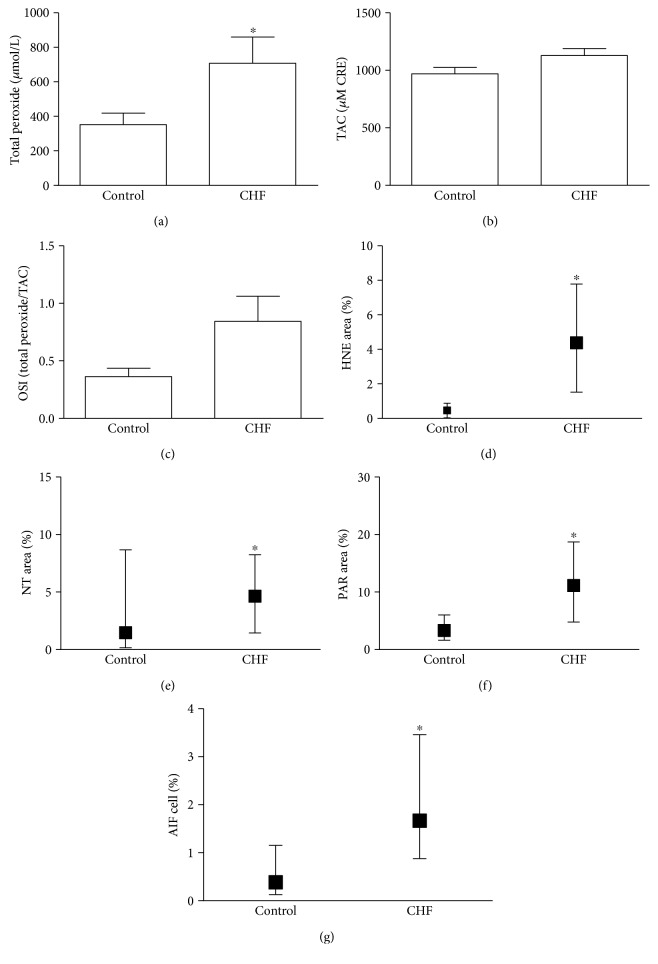
Parthanatos in blood components. (a) Plasma total peroxide level. Data are presented as mean ± SEM. (b) Total antioxidant capacity (TAC) change in TAC values in the CHF and control group. Data are presented as mean ± SEM. (c) Oxidative stress index (OSI) is given as the ratio of total peroxide and TAC. Data are presented as mean ± SEM. (d) Oxidative stress in circulating leukocytes. The percentage of positively labeled cellular area compared to total cellular area was calculated on anti-4-hydroynonenal- (HNE-) immunostained leukocyte smears. Data are presented as median (IQR). (e) Nitrative stress in circulating leukocytes. The percentage of positively labeled cellular area compared to the total cellular area was calculated on antinitrotyrosine- (NT-) immunostained leukocyte smears. Data are presented as median (IQR). (f) PARP activity in mononuclear cells. Immunohistochemical labeling of poly(ADP-ribose) (PAR). The end product of PARP was evaluated as described above. Data are presented as median (IQR). (g) AIF translocation in mononuclear cells. Immunohistochemical labeling of AIF. The percentage of positively labeled nuclei compared to the total number of nuclei was calculated. Data are presented as median (IQR) ^∗^*p* < 0.05.

**Figure 2:**
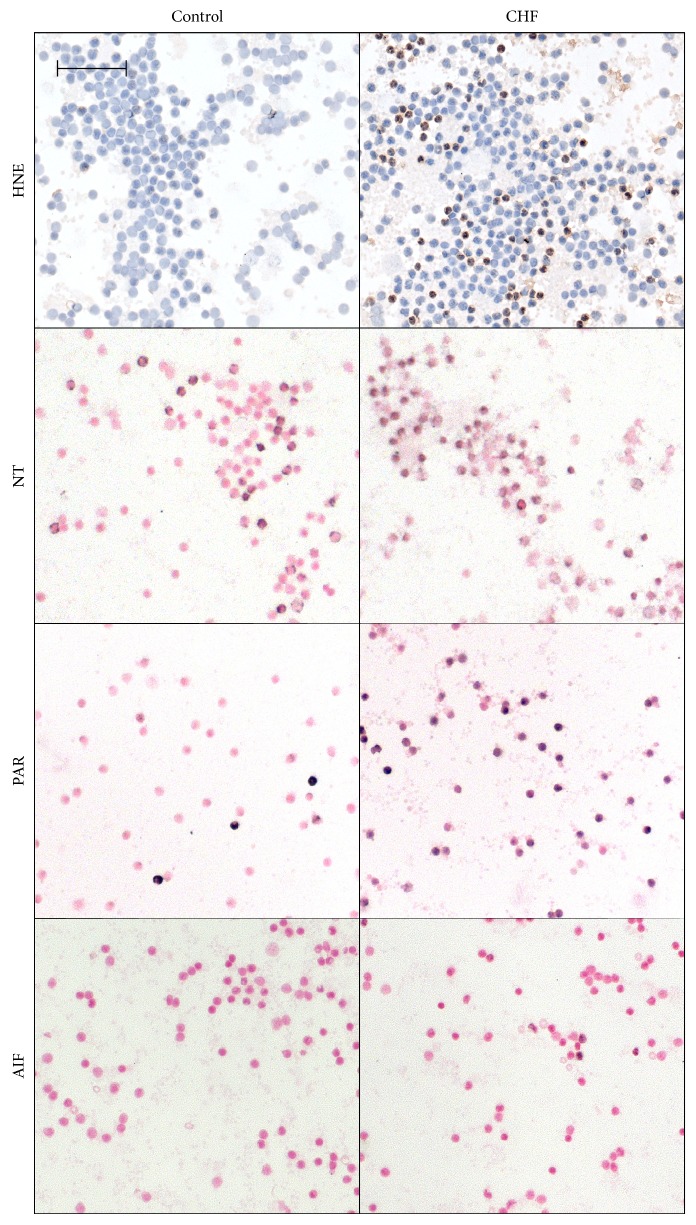
Representative microscopic image of anti-4-hydoxynonenal (HNE), antinitrotyrosine (NT), anti-poly(ADP-ribose) (PAR) stained leukocyte smears, and AIF translocation. The brown-colored (HNE) or black-colored (NT, PAR, and AIF) diamino-benzidine (DAB) represents specific labeling; the blue-colored hematoxylin (HNE) or red-colored Nuclear Fast Red (NFR) (NT, PAR, and AIF) was used for counterstaining. The microscopic images were taken by light microscopy using 40x/0.75 objective; the length of the scale bar is 50 *μ*m. The ratio of positively stained nuclear area is increased in CHF patients' leukocyte smears stained against PAR and AIF.

Plasma PRX level was significantly increased in the CHF group indicating that these patients had more intensive oxidative stress compared to controls. On the other hand, TAC was not significantly elevated in CHF patients (*p* = 0.066). After calculating OSI, we also found a nonsignificant elevation of this parameter among CHF patients (*p* = 0.059). Similarly to PRX, cellular oxidative stress assessed by leukocyte HNE immunostaining intensity was also significantly increased in the CHF group compared to controls. We observed significant (*p* < 0.05) elevation in leukocyte NT staining intensity too, which may suggest an elevated nitrosative environment in CHF. The degree of PAR labeling in circulating mononuclear cells, reflecting the PARP activity in these cells, was significantly elevated in the disease group. Furthermore, in the CHF group, the number of AIF-positive cells was significantly increased compared to the control group (Figures 1 and 2).

### 3.3. Correlation of Oxidative Stress and Signs of Parthanatos with the Indicators of Cardiac Function

**Figure 3:**
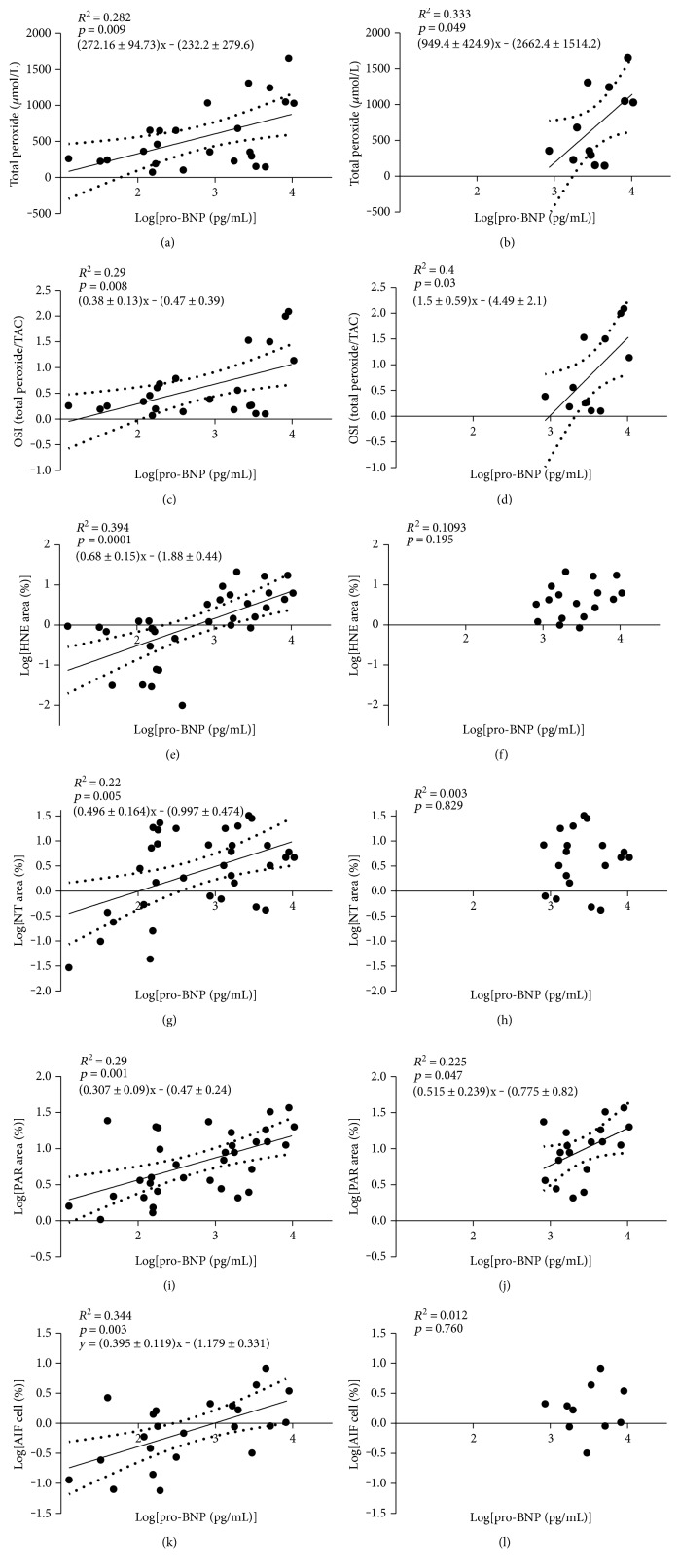
Linear regression analysis of the relationship between oxidative-nitrative stress, PARP activity, or AIF translocation with pro-BNP levels. (a) Linear regression of pro-BNP levels and plasma total peroxide in all subjects. (b) Linear regression of pro-BNP levels and plasma total peroxide in CHF patients. (c) Linear regression of pro-BNP and oxidative stress index in all subjects. (d) Linear regression of pro-BNP and oxidative stress index in CHF patients. (e) Linear regression of pro-BNP and leukocyte lipid peroxidation (4-hydroxynonenal (HNE)) in all subjects. (f) Linear regression of pro-BNP and leukocyte lipid peroxidation (HNE) in CHF patients. (g) Linear regression of pro-BNP and leukocyte tyrosine nitration in all subjects. (h) Linear regression of pro-BNP and leukocyte tyrosine nitration in the CHF patients. (i) Linear regression of pro-BNP and leukocyte PARyalation in all patients. (j) Linear regression of pro-BNP and leukocyte PARyalation in the CHF patients. (k) Linear regression of pro-BNP and leukocyte AIF translocation in all subjects. (l) Linear regression of pro-BNP and leukocyte AIF translocation in the CHF patients. Increased pro-BNP levels are accompanied with increased oxidative stress (PRX, OSI) and PARP activation of circulating mononuclear leukocytes either in the whole study cohort or in the CHF group alone. Lipid peroxidation, tyrosine nitration, and AIF translocation in circulating mononuclear leukocytes however show correlation with the pro-BNP levels only in the total study cohort. Continuous line represents the regression line, while dashed lines show the 95% confidence bends of the best-fit line. *R*^2^: coefficient of determination; *p*: level of significance.

Examining the correlation between oxidative-nitrative stress, PARP activation, AIF translocation, and the used indicators of cardiac function, such as ejection fraction and pro-BNP levels in all study subjects, revealed a significant positive correlation of the latter with all the following parameters: PRX level, OSI, leukocyte HNE, tyrosine nitration, PARylation, and AIF translocation. EF negatively correlated with same parameters: PRX, OSI, leukocyte HNE, tyrosine nitration, PARP activation, and AIF translocation. The observed relationships may imply a negative correlation between cardiac function, oxidative-nitrative stress, PARP activation, and AIF translocation (Table 2, Figures 3(a), 3(c), 3(e), 3(g), 3(i), and 3(k)). Furthermore, AIF translocation also (log[AIF cell %]) positively correlated with LVEDD (*R* = 0.497, *p* < 0.05).

Further, analyzing the same relationships in the CHF group only showed that plasma PRX level, OSI, and PARP activity in circulating leukocytes positively correlate with pro-BNP levels of the chronic heart failure patients. These results suggest a positive correlation of oxidative stress and PARP activation, with the actual clinical appearance of cardiac decompensation reflecting the severity of the chronic heart failure. On the other hand, similar correlation was not observed in case of HNE, tyrosine nitration, or AIF translocation (Table 2, Figures 3(b), 3(d), 3(f), 3(h), 3(j), and 3(l)). In the control group, however, there was no correlation between oxidative stress and the signs of parthanatos and the indicators of cardiac function in the control group (data not shown).

Still, in the CHF group, both LVEDD and LVESD negatively correlated with plasma PRX level and OSI (*R* = −0.819, *p* < 0.01; *R* = −0.838, *p* < 0.01 and *R* = −.0793, *p* < 0.05; *R* = −0.817, *p* < 0.01, resp.) where both LVEDD and LVESD negatively correlated to the presence of ischemic heart disease (*R* = −0.660, *p* < 0.05 and *R* = −0.673, *p* < 0.05, resp.).

### 3.4. Correlation of Oxidative Stress and Signs of Parthanatos with Anamnestic Data and Clinical Parameters

In the whole study cohort, plasma PRX level and OSI positively correlated with the presence of ischemic heart disease (*R* = 0.587, *p* < 0.01 and *R* = 0.573, *p* < 0.01, resp.) and smoking habit (*R* = 0.552, *p* < 0.01 and *R* = 0.525, *p* < 0.01, resp.). Leukocyte nitrative stress (log[NT area (%)]) positively correlated with type 2 diabetes mellitus (*R* = 0.376, *p* < 0.05).

In the CHF group, total plasma peroxide level and OSI positively correlated with the presence of ischemic heart disease (*R* = 0.676, *p* < 0.05 and *R* = 0.687, *p* < 0.05).

### 3.5. Determinants of Oxidative Stress in Chronic Heart Failure

According to our results, plasma PRX levels and OSI in the CHF patients positively correlated both with the pro-BNP levels and the occurrence of ischemic heart disease and negatively with the LVESD and LVEDD diameters. In order to estimate the role of these variables in the development of oxidative stress, multivariate regression was implemented. The analysis showed that serum pro-BNP and IHD are independent determinants of plasma total peroxide level, and together they can explain the 97.7% of its variability. On the other hand, only serum pro-BNP was found to be the independent determinant of OSI (Table 3).

## 4. Discussion

According to the results of both experimental and clinical studies, increased oxidative stress is characteristic for heart failure [3, 4, 15]. In some animal models, a positive correlation between myocardial level of reactive oxygen species (ROS) and ventricular dysfunction was observed [3]. Lipid peroxidation was also shown to be elevated in patients with hypertrophic cardiomyopathy, and the level of oxidative stress was correlated to left ventricular dilation and systolic dysfunction [15]. Our results describing the increased plasma and mononuclear leukocyte lipid peroxidation among patients with CHF and the found correlation of plasma PRX with cardiac performance and pro-BNP levels correspond to and expend these previous findings. Although several experimental data provided evidence for the role of ROS in progression of heart failure, the results of clinical trials examining the possible beneficial effect of antioxidants—as vitamin C, vitamin A, and co-enzyme Q—are controversial. Attenuation of symptoms was reported in some cases; however, in other studies neither the symptoms nor the exercise capacity was improved. On the other hand, it is also worth to point the fact that some drugs already used effectively in the clinical management of heart failure may have antioxidant properties, which may play a role in their therapeutic efficacy [4].

In our study cohort, plasma PRX level was also positively correlated to the occurrence of ischemic heart disease (IHD). The positive correlation with IHD could be observed both in the whole study cohort and in the isolated CHF group. The effect of acute ischemic episode of the heart on oxidative stress was previously examined in patients after acute myocardial infarction with or without cardiogenic shock, and increased oxidative stress was demonstrated in both cases [14, 16]. According to our results, long-term, chronic ischemic environment may also induce further aggravation of plasma PRX levels in CHF patients. On the other hand, this change is also accompanied by increased plasma TAC, resulting to a not significant elevation of OSI.

In our total study cohort, LVEDD and LVESD showed no correlation with PRX; however, in the isolated CHF groups, cardiac dimensions negatively correlated with both total plasma peroxide levels and the presence of ischemic heart disease. The background of the negative correlation found between cardiac dimensions and total peroxide level is not yet clarified. Our observations that IHD among CHF patients is accompanied by elevated plasma PRX, but less increased cardiac diameters, may explain this phenomenon. On the other hand, it is possible that ROS production is exhausted in the progress of excess cardiac remodeling.

Several previous studies demonstrated the elevation of nitrative stress in various forms of acute and chronic heart failure both in experimental models and human biopsies [17]. Protein nitration is one of the characteristic reactions of the potent oxidant peroxynitrite formed in the spontaneous reaction of superoxide and nitric oxide. Increased nitration of sacro/endoplasmic reticulum Ca^++^ ATPase (SERCA) was reported in human hearts of dilated cardiomyopathy. SERCA inactivation by nitration may lead to the reduced function of the ion pump, hence compromising lusitropy as an early event in the development of CHF [18]. Furthermore, overexpression of inducible NO synthase (iNOS) was demonstrated in human hearts with depressed contractility. iNOS-positive patients had larger left ventricular volume and depressed function; however, the preserved NO generation was associated with higher cardiac work [19]. In our study, significant elevation of protein nitration in circulating leukocytes was observed in the heart failure group. Additional pathophysiological alterations may have relevant impact on the creation of nitrative stress. According to our observation, protein nitration correlates with the presence of diabetes mellitus, which corresponds to the previous findings showing that increased glucose concentrations or glucose fluctuations induce the production of nitrogen-derived free radicals and oxidants, involving peroxynitrite, which in turn plays an important role in the pathogenesis of type 1 and type 2 diabetes mellitus and in their complications including cardiovascular morbidities [20].

Experimental studies provided increasing number of evidence of increased oxidative-nitrative stress-induced PARP activation in the development of cardiac dysfunction. Central role of PARP was observed in the pathogenesis of cardiac and endothelial dysfunction in animal models of streptozotocin induced and genetic diabetes mellitus [21]. The animal model of heart failure with chronic aortic partial occlusion was also found to be in association with PARP activation [22]. The elevated activity of the enzyme was also observed in doxorubicin-induced heart failure [23] and endotoxin-induced cardiac depression (*E. coli* lipopolysaccharide (LPS)) [24]. Despite of the high number of experimental publications, fewer human or clinical studies were published. Molnar et al. provided evidence of oxidative stress and PARP activation in 8 human failing heart tissue samples derived from organs that were explanted during heart transplantation. But neither nitrotyrosine staining nor significant translocation of apoptosis-inducing factor was presented [25]. In sepsis-induced human heart failure, significant PARP activation was detected by histological analysis of heart sections and the level of activation correlated to both troponin I and left ventricular systolic stroke work [26]. Poly (ADP-ribosyl)ation in end-stage heart failure after left ventricular assist device implantation was detected in human myocardial biopsies [27]. One of the difficulties of establishing human studies examining the role of any parameter in the development of chronic heart failure is the limited availability of freshly isolated human heart tissue samples. According to our previous results, activation of PARP in circulating leukocytes can be measured in PCI-treated STEMI patients [14]. Moreover, Li et al. found that in human septic shock PARP activity of circulating mononuclear cells was strongly negatively correlated with ejection fraction and showed to be an independent risk factor of myocardial dysfunction [28]. Our present results confirmed the activation of PARP enzyme in human circulating leukocytes derived from patients with chronic heart failure and the level of activation correlated with severity of the disease. These findings may suggest that investigating PARP activation in human circulating leukocytes may serve as an eligible method to describe systemic PARP activation with minimal invasive intervention. It may provide an adequate research or even clinical tool to examine the role of PARP activation in cardiac failure.

According to recent findings, the activation of both heart-hosted and circulating mononuclear cells can be observed in chronic heart failure independently of the etiology of CHF. The consequently evolving sustained pathological chronic inflammation plays an important role in the pathogenesis of the disease. The unbalanced T and B cell activation described by various studies is shown to be associated with cardiomyocyte death, tissue remodeling, and fibrosis that may lead to the progression of the disease [29]. The increased PARP activation in circulating mononuclear cells of CHF patients observed in our study may be involved in the activation of these cells. Therefore, PARP activation of these cells may not just follow the progression of CHF, but they may also contribute to its pathogenesis.

Parthanatos is a new definition based on more detailed results about PARP function in AIF translocation and caspase-independent apoptotic cell death. In a mouse model, heart failure induced by aortic constriction translocation of AIF was attenuated by PARP inhibition [30]. Zhao et al. demonstrated that in experimentally induced congestive heart failure, PARP and AIF protein expression levels were significantly higher in the CHF group than those in the control group [31]. According to our previous results, activation of PARP was followed by the translocation of AIF in PCI-treated STEMI patients [14]. In another human study, increased AIF protein expression and a trend of AIF translocation were observed in atrial tissues following cardioplegia and cardiopulmonary bypass [32]. Furthermore, Molnar et al. found no signs of AIF translocation neither in explanted failing hearts nor in control healthy donor hearts during their heart transplant studies [25]. In our present study, we were able to show increased AIF translocation in circulating mononuclear cells of CHF patients. Moreover, the level of AIF translocation correlated with markers of cardiac function (EF, pro-BNP) and ventricular end diastolic diameter. On the other hand, analyzing the CHF cohort revealed no such correlations.

Our present results together with the previous findings proved the presence of oxidative-nitrative stress-induced PARP activation and its deleterious consequences in chronic heart failure. The finding that these phenomena correlating with the actual cardiac function can be even observed in blood components may help to the better characterization of the actual significance of PARP activation and parthanatos in CHF. Additionally, further examinations may be required to clarify the source of these systemic alterations and their connection to cardiac function. The overall understanding of these changes may also strengthen the concept of PARP inhibitor treatment in CHF raised by several animal model studies. The pharmaceutical inhibition and the genetic deficiency of PARP were previously proven to be beneficial in various types of heart failure. In doxorubicin-induced heart failure of mice treatment with the PARP inhibitor INO-1001 reduced mortality and cardiac depression [33]. The same inhibitor improved cardiac performance in aging animals and also promoted acetylcholine-induced, nitric oxide-dependent vascular relaxation [34]. Furthermore, other PARP inhibitor PJ34 was shown to prevent Ca^2+^ overload and oxidative stress-induced myocardial contractile disturbances in doxorubicin-treated rats [35]. In a rat model of heart failure, PARP inhibition with INO-1001 improved cardiac function after the permanent occlusion of the left anterior descending coronary artery [33]. In a similar mouse model, INO-1001 prevented the decline of cardiac function and attenuated hypertrophy and tissue remodeling [30].

Several contributors of cardiac hypertrophy have been previously identified; among others is angiotensin II, which is recognized as one of the most potent stimulators of cardiac hypertrophy and remodeling. PARP-deficient mice were shown to be protected from angiotensin II-induced cardiac hypertrophy, and it was suggested that PARP contributes to the angiotensin II-induced signaling pathways leading to cardiac hypertrophy and failure [36]. In a spontanenously hypertensive rat model, the chronic inhibition of PARP with L-2286 prevented remodeling, preserved systolic function, and delayed transition of hypertensive cardiopathy to heart failure [37]. In a rat model of chronic heart failure induced by isoproterenol-provoked myocardial infarction, the effect of L-2286 was also investigated. The PARP inhibitor significantly reduced the progression of heart failure by reducing cardiac hypertrophy and interstitial fibrosis [38]. The antiremodeling effect of L-2286 was compared to the efficacy of a widely used angiotensin-converting enzyme (ACE) inhibitor, enalapril, in the same experimental model. The PARP inhibitor was found to decrease the postinfarction myocardial remodeling even more effectively than the enalapril treatment [39].

## 5. Conclusion

Our present study showed that components of parthanatos can be observed in the blood components of patients with chronic heart failure; oxidative-nitrative stress, PARP activation, and AIF translocation also correlate to reduced cardiac function. Moreover, oxidative stress and PARP activation may also indicate the progression of heart failure. The positive correlation between leukocyte PARP activation and pro-BNP may assign the minimal invasive measurement of this parameter to a possible diagnostic tool for heart failure progression monitoring. It may also serve as a potential early detection tool for incipient heart failure. Our data may also legitimate the further investigation of PARP inhibition, as a therapeutic target in chronic heart failure.

## Figures and Tables

**Table 1:** Clinical parameters and anamnestic data of study groups.

	Control	Chronic heart failure	Sign
Number	15	20	
Gender: male/female	13/2	16/4	n.s.
Age (years)	63.3 ± 9.4	68.9 ± 8.0	n.s.
Body mass index (kg/m^2^)	28.9 ± 3.3	27.9 ± 4.9	n.s.
EF (%)	60.0 ± 5.3	24.9 ± 5.9	*p* < 0.01
LVEDD (mm)	48.2 ± 4.6	63.0 ± 11.1	*p* < 0.01
LVESD (mm)	31.4 ± 5.1	54.0 ± 10.6	*p* < 0.01
Pro-BNP (pg/mL)	148.4 (47.9; 178.0)	2338.5 (1475.6; 4597.5)	*p* < 0.01
CRP (mg/L)	5.0 (1.3; 6.1)	2.6 (1.5; 4.9)	n.s.
Smoking (*N*)	1	3	n.s.
Diabetes mellitus (*N*)	2	4	n.s.
Hypertension (*N*)	13	19	n.s.
Ischemic heart disease (*N*)	3	9	n.s.

Statistical analysis: continuous variables are presented as mean ± SD or median (IQR) in case of variables that violated the normal distribution; categorical variables are presented as the number of affected patients in each group. In order to test the possible differences between the study groups, *t*-test or chi-square test was performed. Sign: significance; *N*: number; n.s.: nonsignificant; EF: ejection fraction; LVEDD: left ventricular end diastolic diameter; LVESD: left ventricular end systolic diameter; Pro-BNP: N-terminal probrain-type natriuretic peptide; CRP: C-reactive protein.

**Table 2:** Pearson correlation of oxidative-nitrative stress markers, PARP activity, AIF translocation with ejection fraction, and pro-BNP levels.

		All subjects		CHF patients	
		EF (%)	Log[pro-BNP (pg/mL)]	EF (%)	Log[pro-BNP (pg/mL)]
Total peroxide (*μ*mol/L)	*R*	**−0.458**	**0.531**	−0.245	**0.577**
	*p*	0.028	0.009	0.444	0.049
	*N*	23	23	12	12

TAC (*μ*mol CRE)	*R*	−0.244	0.142	0.226	−0.412
	*p*	0.165	0.424	0.351	0.08
	*N*	34	34	19	19

OSI (total peroxide/CRE)	*R*	**−0.466**	**0.537**	−0.339	**0.630**
	*p*	0.025	0.008	0.291	0.028
	*N*	23	23	12	12

Log[HNE area (%)]	*R*	**−0.682**	**0.628**	0.336	0.331
	*p*	0.00002	0.0001	0.187	0.195
	*N*	32	32	17	17

Log[NT area (%)]	*R*	**−0.368**	**0.497**	−0.213	0.051
	*p*	0.032	0.009	0.381	.836
	*N*	34	34	19	19

Log[PAR area (%)]	*R*	**−0.466**	**0.576**	−0.004	**0.474**
	*p*	0.006	0.000	0.986	0.047
	*N*	33	33	18	18

Log[AIF cell (%)]	*R*	**−.431**	**.472**	0.000	−.169
	*p*	0.035	0.020	0.999	0.620
	*N*	24	24	11	11

Statistical analysis: Pearson correlation was performed between continuous variables. In order to provide the normal distribution of variables that initially violated normality, logarithmic transformation was implemented. *R*: Pearson coefficient; *p*: significance; *N*: number of cases in each calculation. pro-BNP: N-terminal probrain-type natriuretic peptide; TAC: total antioxidant capacity; CRE: copper reducing equivalent; OSI: oxidative stress index; HNE: anti-4-hydroxynoneal; NT: nitrotyrosine; PAR: poly(ADP-ribose); AIF: apoptosis-inducing factor.

**Table 3:** Multivariate regression of the possible determinants of oxidative stress in chronic heart failure.

	Total peroxide (*μ*mol/l) *r*^2^ = 0.977, *p*^A^ < 0.002, *N* = 12			OSI *r*^2^ = 0.978, *p*^A^ < 0.001, *N* = 12		
	*B*	SD of *B*	*p* ^B^	*B*	SD of *B*	*p* ^B^
Constant	−1611.2	713.8	0.087	−2.629	0.927	**0.047**
Log[pro-BNP (pg/mL)]	991.7	154.5	**0.003**	1.402	0.201	**0.002**
IHD	362.4	127.5	**0.047**	0.421	0.166	0.064
LVEDD	26.3	25.7	0.362	0.059	0.033	0.151
LVESD	−55.2	30.2	0.141	−0.101	0.039	0.062

Dependent variable was plasma total peroxide level (*μ*mol/L), while independent variables were plasma pro-BNP, the occurrence of ischemic heart disease (IHD), LVEDD, and LVESD. *r*^2^: coefficient of determination of the model; *p*^A^: significance level of the model; *B*: beta coefficient; *p*^B^: significance level of the effect of each independent variable. OSI: oxidative stress index; pro-BNP: N-terminal probrain-type natriuretic peptide; IHD: ischemic heart disease; LVEDD: left ventricular end diastolic diameter; LVESD: left ventricular end systolic diameter.
