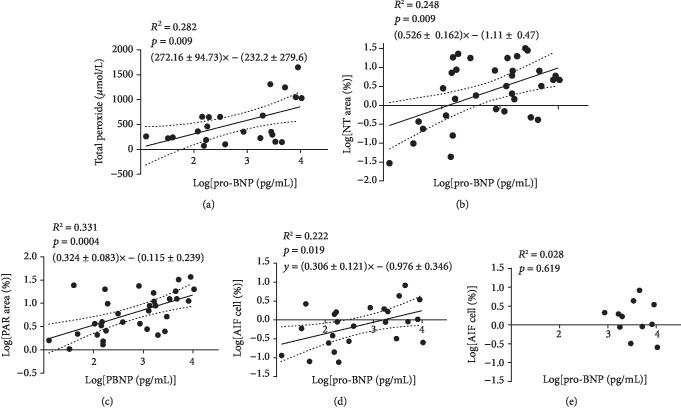# Corrigendum to “Oxidative Stress-Related Parthanatos of Circulating Mononuclear Leukocytes in Heart Failure”

**DOI:** 10.1155/2019/8747486

**Published:** 2019-10-23

**Authors:** Tamás Bárány, Andrea Simon, Gergő Szabó, Rita Benkő, Zsuzsanna Mezei, Levente Molnár, Dávid Becker, Béla Merkely, Endre Zima, Eszter M. Horváth

**Affiliations:** ^1^Department of Physiology, Semmelweis University, Budapest, Hungary; ^2^Heart and Vascular Center, Semmelweis University, Budapest, Hungary; ^3^Institute of Human Physiology and Clinical Experimental Research, Semmelweis University, Budapest, Hungary

In the article titled “Oxidative Stress-Related Parthanatos of Circulating Mononuclear Leukocytes in Heart Failure” [1], incorrect versions of Figures 3(a), 3(g), 3(i), 3(k), and 3(l) were published, as one data point was affected in each curve, due to drift during the data transfer from SPSS 22.0 to GraphPad Prism software. However, these errors do not change any outcome of the study or any further details of the publication. The correct versions of the mentioned figures are shown below:

## Figures and Tables

**Figure 1:**